# Fluid and Electrolyte Disorders in Traumatic Brain Injury: Clinical Implications and Management Strategies

**DOI:** 10.3390/jcm14030756

**Published:** 2025-01-24

**Authors:** Vivie Tran, Jackeline Flores, Meagan Sheldon, Camilo Pena, Kenneth Nugent

**Affiliations:** Department of Internal Medicine, Texas Tech University Health Sciences Center, Lubbock, TX 79430, USA; vivie.tran@ttuhsc.edu (V.T.); jackelinebriggitte@gmail.com (J.F.); meagan.hoover@ttuhsc.edu (M.S.); camilo.pena@ttuhsc.edu (C.P.)

**Keywords:** traumatic brain injury, blood–brain barrier, diabetes insipidus, syndrome of inappropriate antidiuretic hormone secretion, thirst, hyponatremia, hypernatremia

## Abstract

Traumatic brain injuries (TBIs) cause direct central nervous system injury. The presentation depends on the location, the type, and the severity of the injury. Additional injury may develop secondary to compression, the disruption of cerebral perfusion, and changes in sodium levels, resulting in either cellular edema or dehydration. Plasma osmolality (Posm) is a critical parameter influenced by solute concentrations, including sodium, glucose, and urea, and is a relevant concern when considering sodium levels in these patients. While Posm can be calculated using a standard formula, direct measurements via osmometry offer better accuracy. It is essential to differentiate between osmolality and tonicity; the latter refers specifically to effective solutes that drive water movement in the extracellular fluid. Sodium and its anions are effective solutes, whereas urea and glucose have variable effects due to their permeability and insulin dependence. Following TBI, the dysregulation of osmoregulation may occur and affect neurological outcomes. Osmoreceptors in the brain regulate arginine vasopressin secretion in response to changes in effective solute concentrations, with sodium chloride and mannitol being potent stimuli. The regulation of plasma osmolality, typically maintained within ±5% of the 280–295 mOsm/kg H_2_O range, is crucial for homeostasis and relies on antidiuresis and thirst mechanisms. This review narrative underscores the complexities of osmoregulation in the context of TBIs and their clinical implications, particularly concerning the development of conditions such as diabetes insipidus, the syndrome of inappropriate antidiuretic hormone secretion, and abnormal thirst.

## 1. Introduction

Patients with traumatic brain injury (TBI) have complex presentations [1]. Important factors in these presentations include the type of injury, the location of the injury, and the severity of the injury. The initial classification of these patients often uses three items: the Glasgow coma scale, the time needed to recover consciousness, and the time of post-injury amnesia. Factors that influence outcomes include the individual’s age, underlying comorbidities, and access to specialized treatment facilities [2,3]. The consequences of injury can be analyzed as acute events during hospitalization and chronic complications posthospitalization. Additional injury after the initial trauma event likely develops when patients have increased intracranial pressure, increased intracranial bleeding, decreased brain blood flow secondary to hypotension or vasospasm, seizures, and abnormal electrolytes, resulting in cellular dysfunction, i.e., dehydration or edema. Smith et al. reported the outcomes of 151 patients who presented to a trauma center [4]. Patients were classified into two cohorts: hyponatremia with serum sodium less than 135 mEq/L upon admission and non-hyponatremia. Hyponatremia has a significant association with mortality (45.5% died) and is an important predictor of mortality in multiple regression analysis. The American College of Surgeons Trauma Program published best practice guidelines for the management of TBIs in 2024 [1]. The treatment goals recommended in this guideline include serum sodium in the range of 135 to 145 mEq/L and a serum osmolality ≤320 mOsm/kg, and this indicates that routine medical care is critical during the initial hospitalization.

The Centers for Disease Control and Prevention publishes information on the annual number and rate of deaths per 100,000 TBIs in the United States [5]. There were 60,565 TBI-related deaths in 2018 and 60,611 deaths in 2019. The 2019 rate ranged from 1.3 per 100,000 in children aged 5 to 9 to 76.7 per 100,000 in adults 75+ years of age. The adjusted rate was approximately three times higher in men. The rate of unintentional motor vehicle crash deaths was 3.1 per 100,000 for all ages, the rate for unintentional falls was 5.5 per 100,000 for all ages, and the rate of suicide was 7.5 per 100,000 for all ages. The adjusted rates for all ages in these three categories were higher in men than in women. Flores-Sandoval et al. (2024) published a systematic review of studies reporting mortality and disposition in adults with moderate-to-severe TBI [6]. This review included 64 studies published between 1992 and 2022. Mortality was higher in adults older than 60 years of age than in younger adults. In addition, older adults (>65 years of age) were less likely to be discharged home than younger adults and were more likely to be discharged to skilled nursing facilities, inpatient rehabilitation, and palliative or hospice care. These studies indicate that traumatic brain injury occurs in all age groups and can have important outcomes, including death and chronic disability. Consequently, immediate medical and surgical management becomes a priority in all healthcare facilities admitting these patients.

This review discusses the importance of extracellular fluid osmolality and regulation in cellular homeostasis after a TBI and the disruption of the blood–brain barrier in TBIs as part of the pathogenesis of complications. It also analyzes the important electrolyte abnormalities that can develop after TBIs, including diabetes insipidus, the syndrome of inappropriate antidiuretic hormone secretion, cerebral salt-wasting, and primary thirst disorders in these patients. These disorders can cause very abnormal sodium levels and have important effects on cerebral cell volumes and cellular function that need immediate attention during the management of these patients. This review provides a summary resource for clinicians managing these patients in intensive care units and alerts the clinician to the possibility of chronic sodium disorders after the acute management phase in the hospital.

## 2. Methods

PubMed was used for the literature searches in this study. The MeSH terms included brain injury, traumatic; inappropriate ADH syndrome; diabetes insipidus; polydipsia, psychogenic; hyponatremia; hypernatremia; blood–brain barrier; and osmolar concentration. The text word was “cerebral salt wasting syndrome”. These terms were also used in Google and Google Scholar for additional searches. Searches were limited to English language publications with a focus on adults. The reference list from the recovered articles was also reviewed for additional articles.

## 3. Volume and Osmolality Control in Traumatic Brain Injury

Plasma osmolality (Posm) is primarily determined by the concentrations of solutes in plasma, including sodium, glucose, and urea. Before considering its calculation, it is important to understand the difference between osmolality and tonicity. Osmolality refers to the total concentration of all dissolved particles (solutes) in a solution, regardless of their ability to cross cell membranes. Tonicity, however, refers specifically to the effective osmotic pressure exerted by solutes that cannot freely cross cell membranes. These are the solutes that drive water movement between fluid compartments. Sodium and its accompanying anions are effective solutes; urea and glucose have variable effects depending on their permeability and insulin dependence, respectively [7].

Plasma osmolality can be calculated using a standard formula, though direct measurement via osmometry is more accurate. The standard formula is given as follows:

Posm (mmol/kg) = 2 × serum Na^+^ (mEq/L) + glucose (mg/dL)/18 + BUN (mg/dL)/2.8

However, to determine effective osmolality (tonicity), which reflects the osmotic pressure driving water movement across cell membranes, urea should be excluded because it readily crosses cell membranes:

Effective Posm (mmol/kg) ≈ 2 × serum Na^+^ (mEq/L) + glucose (mg/dL)/18

Following TBI, the dysregulation of osmoregulation can significantly affect neurological outcomes. Osmoreceptors in the brain regulate arginine vasopressin (AVP) secretion in response to changes in effective solute concentrations. Sodium chloride and mannitol are potent stimuli for AVP release. The regulation of plasma osmolality, typically maintained within ±5% of the 280–295 mOsm/kg H_2_O range, relies on antidiuresis and thirst mechanisms. This section of the review underscores the complexities of osmoregulation in TBIs and their clinical implications [8].

The law of osmosis explains water movement across semi-permeable membranes—those allowing water but not solutes to pass. When a semi-permeable membrane separates into two compartments, water molecules will diffuse into the area of higher solute concentration. Thus, an increase in free water in the ECF will prompt water movement across the membrane, resulting in intracellular expansion, while a decrease in free water in the ECF will cause water to shift from the ICF to the extracellular space. Increases in volume also increase the extrinsic compression on cerebral cells in a closed space, and decreases in volume likely distort neural tracts and function [7].

Osmoreceptors in the brain detect solute concentrations and modulate the secretion of arginine vasopressin (AVP) from the posterior part of the pituitary gland. Only effective solutes stimulate AVP secretion; studies have demonstrated that solutes like sodium chloride and mannitol are significantly more effective than urea or glucose in this process [8]. Plasma osmolality is rigorously regulated within a ±5% tolerance, with the normal range of 280–295 mOsm/kg, driven by tonicity rather than total osmolality. Two primary mechanisms maintain effective Posm: the pituitary secretion of AVP, which promotes antidiuresis, and the thirst response, which compensates for water losses. Each mechanism is governed by distinct regulatory processes.

The neurohypophysis constitutes the location where AVP is synthesized in the paraventricular and supraoptic nuclei, subsequently transported down the pituitary stalk, and then released into capillaries from neuron terminals in the posterior part of the pituitary gland. Damage to this region, such as from TBIs, may not lead to diabetes insipidus if the AVP-producing cell bodies in the hypothalamus remain intact since AVP can be released from higher hypothalamic areas [7].

The vasopressin gene encodes a 154-amino-acid prohormone that undergoes cleavage into three peptides: neurophysin, AVP at the amino terminus (comprising 9 amino acids), and copeptin at the carboxy terminus. Copeptin is released in equimolar amounts with AVP; due to its larger size and longer half-life, it serves as the preferred surrogate marker for AVP secretion. Once in circulation, AVP interacts with three primary receptor subtypes: V1a, V1b, and V2. The kidneys are exquisitely sensitive to changes in plasma AVP, and urine volume is inversely proportional to AVP concentration. Specifically, lower AVP levels result in increased urine excretion, and in cases of complete central diabetes insipidus, urine excretion rates may reach up to 1000 milliliters per hour. Physiological urine concentrations occur with plasma AVP levels in the range of 0.5 to 5.0 pg/mL. Although baroreceptors are less sensitive to AVP levels, they also stimulate its release in response to changes in blood pressure and blood volume exceeding 10–15% [9]. Thirst correlates linearly with fluctuations in plasma osmolality, but this response is not simultaneous with AVP secretion. A higher threshold for plasma osmolality, typically 5–10 mOsm/kg, is required to stimulate thirst compared to AVP release, thus positioning AVP as the primary osmotic regulator [10,11].

The thirst center is anatomically distinct from AVP-producing neurons and has been identified by positron emission tomography studies in the anterior midcingulate cortex; however, both centers receive input from osmoreceptors [12]. The primary osmoreceptors are located in the anterior wall of the third ventricle, extending from the anterior commissure to the organum vasculosum of the lamina terminalis (OVLT), collectively referred to as the AV3V area. The OVLT, positioned outside the blood–brain barrier, allows for the rapid sensing of changes in plasma osmolality, which stimulates both thirst and AVP secretion [13].

Traumatic lesions affecting AVP neurons result in decreased AVP secretion, leading to diabetes insipidus characterized by polydipsia and polyuria. Conversely, a lesion in the anterior hypothalamus that damages osmoreceptors can lead to both a loss of AVP secretion and an absence of thirst and produces a disorder called adipsic diabetes insipidus. These patients are often chronically hyperosmolar, indicating that AVP secretion alone is insufficient to maintain water homeostasis. Diagnosing this condition can be challenging, as patients are unable to secrete AVP in response to hyperosmolar stimuli but can respond to hypovolemic stimuli. This combination reflects the selective dysfunction of osmoreceptors, which is consistent with the understanding of various baroreceptor pathways within the brain [14,15].

## 4. Blood–Brain Barrier Disruption

**Figure 1:**
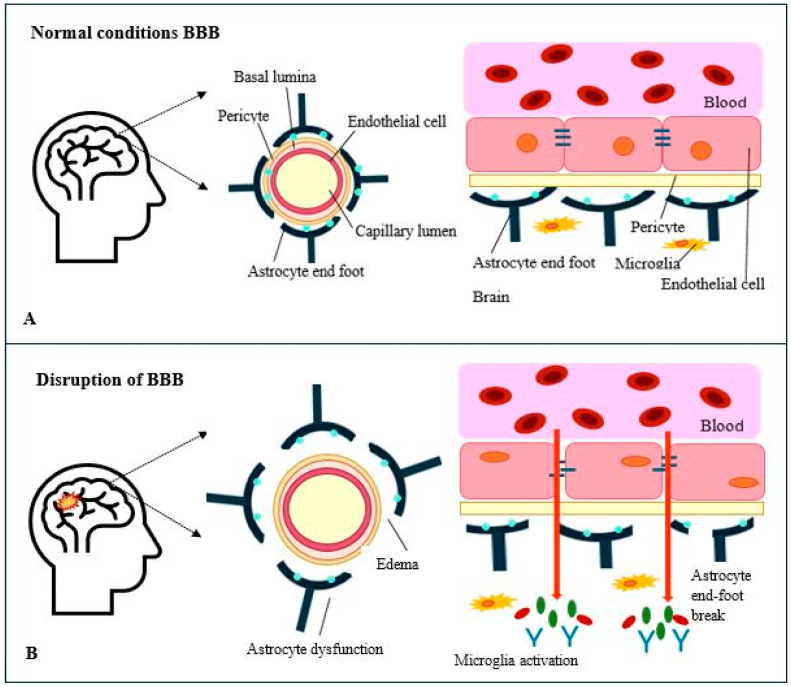
Schematic representation of blood-brain barrier (BBB). Panel **A** shows in physiologic conditions the different components including astrocyte end foot processes, pericytes, Aquaporin-4 (AQP4) channels, and endothelial cells. Panel **B** shows part of the findings when disruption of the BBB occurs with traumatic brain injury leading to development of cerebral edema, passage of substances presumably plasma, platelets, red and white cells, fibrinogen, immunoglobulins, among others, and the role of the microglia activation in a pro-inflammatory state. Created by JF December 2024.

The brain is protected by the blood–brain barrier (BBB) formed by endothelial cells surrounded by cerebral pericytes and the foot processes of astrocytes [16,17]. The cerebral spinal fluid volume is 20 to 25 mL, the subarachnoid space volume is approximately 120 mL, and the interstitial fluid volume in the brain is approximately 250 mL [18]. Electrolyte concentrations in these fluids include sodium at 135 to 150 mmol/L, potassium at 2.7 to 3.9 mmol/L, and chloride at 110 to 125 mmol/L. Protein levels range from 15 to 60 mg/dL. The composition of the fluids is tightly controlled by tight junctions in the endothelial cells and the transport processes across these cells (Figure 1).

Traumatic brain injury causes a significant disruption of the BBB, which can contribute to secondary injury mechanisms. This disruption typically occurs within hours of the initial injury and can persist for years in some patients. The breakdown of the BBB allows plasma with serum proteins, such as fibrinogen and immunoglobulins, leukocytes, erythrocytes, and platelets, to extravasate into the fluid around the brain parenchyma, a phenomenon observed in both acute and chronic cases of TBI [19]. The extent of the injury associated with blood–brain barrier disruption depends on the extent of disruption, the development of fibrin clots and microthrombi in the region, and increases in interstitial fluid pressure.

The disruption of the BBB contributes to the development of cerebral edema and injury through two primary mechanisms. The first is vasogenic edema, characterized by the accumulation of fluid in the perivascular space. This fluid accumulation can lead to alterations in cerebral blood flow and increased intracranial pressure. The second mechanism is cytotoxic edema, which involves the activation of ion channels that facilitate water influx into intracellular spaces, further compromising the integrity of the BBB. If not promptly addressed, these processes can result in irreversible tissue damage and cell death, significantly contributing to the high mortality rates associated with severe TBIs [19]. In most cases, the extent and location of the disruption of the BBB are unknown, but these disruptions likely contribute to progressive brain injury.

In summary, injury to the hypothalamus and pituitary glands can cause changes in the secretion of AVP and thirst responses. Both increases and decreases in these two homeostatic processes can lead to abnormal sodium concentrations and changes in intracellular and extracellular fluid volumes, resulting in additional cellular injury. The disruption of the BBB also contributes to fluid accumulation at sites of injury. Increases in fluid volume increase the pressure in the closed space in the cranium and can potentially cause herniation, and decreases in volume likely distort neural structures and decrease functions.

## 5. An Overview of Cellular Injury Associated with Changes in Sodium

Hypotonicity, beyond causing cellular swelling, can lead to neurological dysfunction through a cascade of events involving altered neuronal excitability, impaired synaptic transmission, and the disruption of the BBB [20,21]. These effects are not solely attributable to changes in cell volume but include the broader pathophysiological consequences of osmotic stress on the complex integrated function of the brain, including the astrocytic AQP4 channels that regulate water movement across the BBB. Dysfunction in this system can lead to cerebral edema. Similarly, hypertonicity, while primarily causing cellular shrinkage, can lead to cerebral hemorrhage from brain shrinkage and the tearing of the meningeal vasculature. The resulting dehydration can also cause a disruption in the BBB and resultant osmotic imbalance. These complications may present as neurological symptoms and also more severe outcomes, such as herniation and hemorrhage. The initial cellular response of regulated sodium and potassium transport is ultimately insufficient to fully counter osmotic stress, leading over time (approximately 48 h) to the accumulation of organic osmolytes; however, this compensatory mechanism may be overwhelmed in cases of severe or rapid osmotic changes. The clinical presentation depends on the rate of osmolar stress, and children and women have greater sensitivity to this stress. In addition, the brain’s response differs in hyper- vs. hypo-osmolar conditions, impacting the timing and severity of complications. The most important sodium disorders are reviewed in the following sections.

## 6. Diabetes Insipidus

### 6.1. Etiology

Diabetes insipidus (DI) is a hormonal disorder that results in large volumes of dilute urine. There are several clinical disorders associated with polyuria; these include central and nephrogenic DI, primary polydipsia, and rarely gestational DI. Most central diabetes insipidus (CDI) cases occur after the destruction of vasopressinergic neurons by TBIs, neoplasms, neurosurgical procedures, or autoimmune inflammation involving AVP-secreting neurons [22]. Nephrogenic diabetes insipidus (NDI) is the result of the resistance of the kidneys to AVP due to mutations in the gene-encoding AVP receptor 2 (*AVPR2*) or aquaporin 2 (*AQP2*), adverse drug effects, or electrolyte disorders [23]. Drug effects are most commonly caused by lithium or tricyclic antidepressants. Primary polydipsia is characterized by excessive fluid intake that leads to polyuria despite intact AVP secretion and an appropriate antidiuretic renal response [23]. Gestational DI develops secondary to the degradation of ADH, which is caused by the enzyme vasopressinase. All disorders with DI result in water diuresis due to the inability to concentrate in urine. 

### 6.2. Epidemiology

Diabetes insipidus is a rare disorder and has a prevalence of 1 in 25,000 when all causes are considered. It can present at any age, and there is no gender predisposition. Central diabetes insipidus can be acquired or hereditary and occurs in 20% of moderate or severe cases of TBI and in 15% of nontraumatic subarachnoid bleeds [22]. The predominant manifestations of DI include hypotonic polyuria with a urine output of greater than 50 mL/kg in 24 h and polydipsia with fluid intake greater than 3 L/day. In pregnancy, CDI may develop secondary to lymphocytic hypophysitis [22]. In addition, pre-existing partial CDI in pregnant women may become more apparent due to the metabolic effects of placental cysteine aminopeptidase, which is an enzyme that increases the metabolic clearance of oxytocin and AVP [22]. Therefore, in patients with TBI and DI, the possibility of DI secondary to causes other than TBI should be considered.

Traumatic brain injury commonly occurs following motor vehicle accidents, assaults, or falls. This injury can cause swelling around the hypothalamic–pituitary axis or direct damage to the paraventricular neurons, supraoptic hypothalamic neurons, the pituitary stalk, and axon terminals in the posterior part of the pituitary gland. These abnormalities can be transient if the supraoptic and paraventricular neurons form new vascular connections or can become permanent [24]. Transient DI is often seen with transsphenoidal surgery for pituitary tumors or sella/suprasellar lesions. When transient, the post-traumatic DI can last a few days to a few weeks and is associated with a high mortality rate, particularly when it occurs very early after a TBI [25].

### 6.3. Diagnosis

The diagnosis of DI is confirmed by 24 h urine collection measuring urine volume and osmolality levels and the measurement of plasma osmolality, sodium levels, and copeptin levels. Additional investigation with hypertonic saline administration or arginine stimulation testing may be needed to make this diagnosis. Plasma osmolality < 280 mOsm/kg and sodium levels < 135 mEq/L indicate primary polydipsia. Plasma osmolality > 280 mOsm/kg and sodium levels > 147 mEq/L suggest CDI or NDI. To then differentiate between CDI and NDI, copeptin levels should be measured. Copeptin ≤ 4.9 pmol/L occurs with CDI, while levels ≥ 21.4 pmol/L indicate NDI. If both plasma sodium and osmolality levels fall within the normal range, more specialized testing may be necessary. The water deprivation test has been the standard used to diagnose DI for many years; however, it has a low diagnostic accuracy of around only 70%. Alternatively, basal copeptin levels, hypertonic saline infusion, and arginine stimulation testing all have significantly higher sensitivity and specificity. Copeptin levels taken before water deprivation or stimulation tests that exceed 21.4 pmol/L have a 100% sensitivity and specificity for the diagnosis of NDI [23]. The hypertonic saline test can be used to differentiate patients with primary polydipsia from patients with CDI with a 93% sensitivity and 100% specificity [23]. This test is performed by infusing 250 mL of 3% hypertonic saline over 15 min, followed by infusing 0.15 mL of 3% saline/kg/minute with the measurement of copeptin and sodium levels every 30 min. With arginine stimulation testing, copeptin levels measured after arginine infusion had a 92% sensitivity and 93% specificity, distinguishing central DI from primary polydipsia [23]. Lastly, if CDI is diagnosed, magnetic resonance imaging (MRI) is needed to investigate the etiology. The classic MRI findings in CDI include the loss of the posterior pituitary bright spot, which represents the depletion or absence of stored AVP [26]. However, the loss of a bright spot may also occur with age. The second most common MRI abnormality is thickening or enlargement of the pituitary stalk [25]. Pituitary stalk thickening can also occur with small craniopharyngiomas, lymphocytic hypophysitis, infiltrative disorders, autoimmune DI, and metastases, such as germinoma in children and young adults [25]. 

### 6.4. Treatment

Desmopressin is the treatment of choice for CDI and gestational DI and can be administered orally, intranasally, subcutaneously, or intravenously. Oral desmopressin doses range from 0.05 mg to 0.8 mg (in divided doses) per day. The oral form has a lower potency than the nasal form because only about 5 percent is absorbed from the gut [23]. Nasal doses range from 10 mcg to 20 mcg per day. Intravenous or subcutaneous administration is used for acute DI, which often occurs after TBI or neurosurgical surgery. Nephrogenic DI requires the discontinuation of all culprit medications and the use of a renal diet to prevent hypernatremia [27]. The treatment of primary polydipsia is aimed at regulating water intake with the possible additional use of antipsychotic medications. Chronic alcohol consumption, particularly beer, with concurrent use of desmopressin is not recommended due to the risk of hyponatremia and alcohol-induced seizures. Patients with TBI and DI need attention for fluid intake, urine output, and sodium levels to maintain optimum electrolyte levels and serum tonicity.

The natural history of this disorder following TBI is variable. It may resolve and make any ongoing treatment unnecessary and potentially dangerous with overhydration. Please see Table 1 for summary details.

## 7. Syndrome of Inappropriate Antidiuretic Hormone Secretion

### 7.1. Epidemiology in Traumatic Brain Injury

The syndrome of inappropriate antidiuretic hormone secretion (SIADH) is characterized by the excessive release or action of the antidiuretic hormone (ADH), resulting in water retention and consequent hyponatremia. This disorder typically arises from injuries affecting the hypothalamus and posterior pituitary glands, where ADH is produced and secreted [28]. The renal tubules, particularly the collecting ducts, also contribute to this syndrome by responding inappropriately to ADH levels [29]. Furthermore, the ectopic production of ADH, notably in cases of small-cell lung cancer, is a well-recognized cause of SIADH [30]. In the context of TBI, there are limited data regarding the specific sites of injury associated with SIADH. One case report suggests that chronic hyponatremia resulting from SIADH following TBI may reflect damage to the pituitary stalk or posterior pituitary gland, although no structural abnormalities were observed in computed tomography (CT) or MRI in that case [31].

The frequency of SIADH in patients with TBI remains inadequately addressed in the existing literature. Chendrasekhar et al. found that among 310 patients with severe TBI, 32 patients (10%) were diagnosed with SIADH [32]. Other research has also identified SIADH as a significant contributor to hyponatremia in these patients. For example, Born et al. reported that in a cohort of 109 patients with severe TBI, 36 individuals (33%) presented with hyponatremia attributed to SIADH [33]. Similarly, Agha et al. studied 102 patients with moderate-to-severe TBI and found that 14 patients (13.7%) had hyponatremia, with 13 cases due to SIADH and one attributed to the cerebral salt-wasting syndrome (CSWS) [24].

In contrast, Lohani and Devkota observed a different incidence; among their 298 patients with TBI, 50 (16.8%) presented with hyponatremia. Thirty-seven of these patients improved with sodium supplementation alone and did not meet the clinical criteria for SIADH; thirteen required additional sodium retention therapy and were diagnosed with CSWS based on clinical symptoms. The authors highlighted a significant lack of clarity in the literature regarding the mechanisms underlying hyponatremia in TBIs, citing variability in the reported rates for SIADH, CSWS, and hypopituitarism. They recommended more research that incorporates evaluations of blood volume and hormonal assessments to elucidate the causes and frequencies of hyponatremia following a TBI [34].

### 7.2. Clinical Presentation

The clinical presentation of SIADH in patients with TBI depends on the type of trauma and the Na^+^ concentration. In a study with 298 patients with TBI, 50 individuals (16.8%) presented with hyponatremia during hospitalization. The incidence varied by TBI type; 47.9% of the patients had cerebral contusions, 34.8% had acute subdural hematomas, 25% had acute epidural hematomas, and 15.9% had chronic subdural hematomas. Most cases of hyponatremia occurred within three days of injury, with a second peak observed after eight days. The average serum sodium level at the time of initial treatment was 133.7 ± 0.4 mEq/L. Although specific symptoms were not detailed, it was noted that hyponatremia can present with a spectrum of symptoms, ranging from mild manifestations, such as nausea and headaches, to severe complications, such as seizures and coma [35]. In addition, the study found that hyponatremia was associated with prolonged hospital stays and worse clinical outcomes in patients with TBI. There was no significant difference in the initial Glasgow coma scale scores between patients who developed hyponatremia and patients who did not. The need to distinguish between SIADH and CSWS in patients with TBI was emphasized as treatment approaches differ. Among the 50 hyponatremic patients, 37 responded positively to sodium supplementation alone, while 13 required additional hydrocortisone treatment for persistent natriuresis. The precise mechanisms underlying hyponatremia following a TBI remain unclear and are likely multifactorial, indicating the need for more studies incorporating blood volume and hormonal evaluations to better understand the etiology of hyponatremia in these patients [35].

### 7.3. Diagnosis

Diagnosing SIADH requires specific criteria that evaluate osmolality, urinary concentrations, and volume status while excluding other potential causes. Key diagnostic criteria include the reduced osmolality of extracellular fluid, i.e., a plasma osmolality below 275 mOsm/kg, and inappropriate urine concentration, i.e., urine osmolality above 100 mOsm/kg, despite normal renal function. Patients with SIADH are clinically euvolemic without fluid overload or dehydration and have elevated urinary sodium excretion (greater than 30 mEq/L) under normal salt and water intake. Supporting criteria for SIADH diagnosis include low serum uric acid levels (below 4 mg/dL), low blood urea nitrogen (BUN below 10 mg/dL), and uncorrected hyponatremia upon volume expansion, with improvement following fluid restriction. In some cases, a water load test may be used; abnormal results, i.e., excreting less than 80% of a 20 mL/kg water load within four hours, further support SIADH diagnosis. Elevated plasma AVP levels in the presence of hypotonicity and clinical euvolemia also support this diagnosis [36].

Excluding other causes of euvolemic hyponatremia, such as hypothyroidism, glucocorticoid deficiency, or medications that stimulate AVP release, is critical to confirm the diagnosis of SIADH. Fractional excretion of uric acid greater than 12% can further distinguish SIADH from hypovolemic hyponatremia. This diagnostic approach typically includes comprehensive laboratory testing, the assessment of volume status, and a water restriction trial. In complex cases, measuring AVP or copeptin levels, along with conducting water load tests, may be necessary. In general, fulfilling core criteria in a euvolemic patient while ruling out other causes is sufficient for diagnosing SIADH [36].

### 7.4. Treatment

The incidence of hyponatremia in patients with TBI varies across studies. According to a Moro study (2007), the incidence was 16.8%, with 50 out of 298 patients with TBI presenting with hyponatremia. This condition was linked to longer hospital stays and poorer clinical outcomes. Among the hyponatremic patients, 37 improved with sodium supplementation alone, and 13 required additional treatment with hydrocortisone due to persistent natriuresis. The administration of hydrocortisone significantly decreased both sodium excretion and urine volume, indicating its effectiveness in managing hyponatremia [35].

Rajagopal et al. (2017) reported an incidence of hyponatremia of 13.2% in patients with TBI (198 out of 1500). Traumatic subarachnoid hemorrhage was the most frequent abnormality on admission CT scans in patients who developed hyponatremia. The authors noted that early treatment with fludrocortisone significantly reduced the length of hospital stays for affected patients, and they proposed a management protocol for hyponatremia in patients with TBI that used fludrocortisone early in cases accompanied by natriuresis. This approach aims to simplify treatments by eliminating the need to distinguish between SIADH and CSW, potentially streamlining patient care [37].

For all patients with SIADH, the close monitoring of serum sodium is crucial, particularly during the first 24–48 h of treatment, to prevent overly rapid corrections that could lead to osmotic demyelination syndrome. In general, the sodium correction rate should remain below 8–12 mEq/L within 24 h or 18 mEq/L over 48 h; in patients at high risk of osmotic demyelination, even slower correction rates (e.g., below 8 mEq/L in 24 h) are recommended. Hyponatremia and hypo-osmolality can be treated with hypertonic saline, isotonic saline, water restriction, loop diuretics, urea, or vaptans [38]. Personalized treatment based on the severity of hyponatremia, symptoms, and patient risk factors is essential for safe and effective management [28]. These patients are at risk of developing osmotic demyelination syndrome, and some patients develop this even with normal sodium levels.

The net effect of this disorder on fluid balance and sodium levels will depend on residual ADH levels if anything. In addition, the possibility of cerebral salt-wasting syndrome should be considered as an alternative explanation for hyponatremia. Please see Table 2 for the summary details.

## 8. Cerebral Salt-Wasting Syndrome

The cerebral salt-wasting syndrome (CSWS) is another important cause of hyponatremia in patients with TBI and is distinct from SIADH [32]. While both conditions can lead to hyponatremia, CSW is characterized by hypovolemia, whereas SIADH typically presents with euvolemia or mild hypervolemia. Chendrasekhar et al. found that in 310 patients with severe TBIs, 125 (40%) developed CSW, and 32 (10%) developed SIADH. This suggests that CSW may be more common than SIADH in patients with TBI. Patients with CSW had significantly worse outcomes, including higher injury severity scores, longer hospital and ICU stays, more ventilator days, and lower survival rates than patients with TBI without CSW [32]. Distinguishing between CSW and SIADH is crucial for appropriate treatment. Both disorders may initially be treated with hypertonic saline, but patients with CSWS often require additional volume expansion and sometimes mineralocorticoid therapy (e.g., fludrocortisone) for persistent natriuresis [39].

The management of patients with TBI and sodium disorder can be challenging, and it may be difficult to determine if the abnormal sodium levels reflect a clinical syndrome associated with TBI or reflect errors in management. Both hyponatremia and hypernatremia have been associated with poor outcomes in patients with TBI. Hyponatremia is recognized as a significant independent factor associated with adverse neurological outcomes in patients with TBI. In one study, hyponatremia occurred in 13.2% of patients with TBI and was associated with higher 6-month mortality (23.5%) than patients with normal sodium levels (19.9%) [40]. A recent study identified an L-shaped relationship between sodium levels and in-hospital mortality in patients with TBI, with a critical threshold of 144.1 mEq/L. Patients with hypernatremia (sodium levels greater than 145 mEq/L) had a 2.17-fold increased risk of in-hospital death compared to those with normal or low sodium levels, even after controlling for potential confounding variables. This indicates that hypernatremia is independently linked to an increased risk of mortality in patients with TBI, as discussed in the section on DI [41]. In addition, another study reported that 36.9% of patients with severe TBI developed hypernatremia, which was also independently associated with early mortality. The findings indicate that mortality rates are high in patients with severe hypernatremia and indicate that clinicians must not overcorrect hyponatremia during the management of both SIADH and CSWS [42].

The development of hyponatremia can have profound consequences with changes in both osmolality and cell volume. Management requires careful attention to the changes in sodium levels, and rapid increases can cause osmotic demyelination syndrome; this complication has also developed in cases with normal sodium levels [43,44]. In addition, patient behavior during management, especially increased fluid intake, which is not accurately recorded, can influence sodium levels. Please see Table 3 for the summary details.

## 9. Thirst in Traumatic Brain Injury

The initial evaluation of patients with TBI usually focuses on the level of consciousness and neurological status or injuries/deficits. These injuries can cause psychiatric disorders, which, in turn, can have important effects on sodium levels. This leads to the difficult question of whether the initial disorder involved thirst and fluid intake or abnormal antidiuretic hormone levels.

### 9.1. Site of Injury

Normal thirst responses depend on plasma osmolality and the oropharyngeal region that plays a role in the secretion of vasopressin [45,46]. Zimmerman et al. found that the subfornical organ (SFO) is involved in the anticipatory regulation of thirst, which allows drinking behavior to be modified [47]. In conditions such as polydipsia, the exact cause is still uncertain and is probably multifactorial. Available data suggest that it could be due to hypersensitivity, the effects of vasopressin, increases in the activity of dopamine, or even a defect in osmoregulation. Thirst-center stimulation in response to high dopamine levels with increased water intake consumption can counteract the effects of some psychotropic medications, and changes in the feedback regulation of the hypothalamic–pituitary axis in chronic polydipsia have also been described [48].

Studies have revealed changes in the osmotic set point for the release of vasopressin in patients with schizophrenia, and these are increased during acute episodes of psychosis. In addition, vasopressin dysfunction is present due to hippocampal impairment in the regulation of vasopressin and the hypothalamic–pituitary–adrenal axis, which suggests that hippocampal impairment in subjects who have TBIs increases the risk of developing psychogenic polydipsia [49,50].

### 9.2. Epidemiology

The relationship between thirst and changes in sodium has been studied, especially in those with psychogenic polydipsia, which includes a variety of psychiatric conditions, such as bipolar disorder, psychotic depression, and, more commonly, schizophrenia [51]. Patients with psychogenic polydipsia have a water intake greater than the kidneys’ capacity to excrete it and drink up to 1 L/h, resulting in hyponatremia due to a combination of poor solute consumption and excessive water consumption [52].

Mercier-Guidez et al. found that 11–20% of patients with chronic schizophrenia have psychogenic polydipsia with an intake of 5–15 L per day; these patients were symptomatic when sodium levels varied in the range of 106–114 mEq/L [53]. Current data support the idea that TBI is a risk factor for schizophrenia, especially in patients with genetic susceptibilities to psychosis [54]. Conversely, adult patients who had TBI at an older age without any family history of psychiatric disorders can also develop schizophrenia [51]. A study conducted by Trivedi and his team found that hospitalized patients due to TBI have a 2.2 times greater likelihood of schizophrenia, especially in men. Greater risk is present in patients with bipolar disorders, anxiety disorders, personality disorders, substance abuse, intellectual disability, and young age [55].

### 9.3. Clinical Presentation

Symptoms suggesting hyponatremia in patients with psychiatric disorders include headache, fatigue, weakness, irritability, confusion, nausea, vomiting, and muscle cramps [56]. The presentation depends on how low the sodium level is and its rate of development. These symptoms are uncommon except in patients with an intake greater than 10 L daily because they have reached their capacity to dilute urine and have developed complete vasopressin suppression [57]. Attention should also be given to patients with urinary incontinence, including enuresis, as this can suggest polydipsia.

### 9.4. Diagnosis

Part of the workup when addressing abnormalities regarding excessive thirst requires ruling out other causes of polydipsia. Imaging studies are potentially useful since the thirst center can be affected by infiltrative diseases [58]. The context of the patient will suggest the possible etiology; for example, psychiatric patient behavior with increased fluid consumption due to delusional states or obsessive-compulsive tendencies or the use of psychotropic medications with anticholinergic side effects can lead to signs and symptoms of episodes with hyponatremia [56,59]. Hospitalized patients with alcoholism or eating disorders can use water to obtain pseudosatiety.

The water restriction test is the gold standard for the diagnosis of psychogenic polydipsia. In this test, a Posm higher than 295 mOsm/kg causes a maximal renal response to increased vasopressin levels. Before the test, patients usually have very diluted urine with an osmolality lower than 100 mOsm/kg and low levels of vasopressin. After the test is carried out, patients have concentrated urine with an osmolality greater than 600 mOsm/kg and high levels of vasopressin [57].

### 9.5. Treatment

The onset and severity will guide the treatment approach; patients with acute symptomatic and severe hyponatremia require treatment as described previously in the other sections. Fluid restriction should be guaranteed in patients with psychogenic polydipsia and hyponatremia [58]. Non-pharmacological management should include reinforcement schedules and fluid restriction. In monitored settings like hospitals or nursing homes, management should include measuring diurnal weight and enforcing short fluid restriction periods (between 1 and 3 h); acute increases in weight greater than 5–8 kg put the patient at risk of water intoxication [60]. Pharmacological medications can include antipsychotic medications, e.g., risperidone and clozapine, and other medications, such as angiotensin-converting enzyme inhibitors, beta-blockers, and alpha-blockers, and have provided beneficial results in these patients [61,62,63,64]. However, their use has been reported in case studies, and no large trials or clear guidelines are available. Long-term management requires behavioral management and close vigilance by health professionals due to the likelihood of relapsing behavior.

Please see Table 4 for summary details.

## 10. Conclusions and Prospects

Patients with TBIs have complex presentations that depend on the type and severity of the injury and other trauma-related injuries [1]. They likely will need evaluation by a neurosurgeon, a trauma surgeon, a critical care specialist, and a neurologist. A careful review of medical history to determine prior medical disorders and current drug use is essential but may be difficult depending on circumstances. The initial injury may be complicated by the development of additional intracranial bleeding, intracranial hypertension, decreased cerebral perfusion, and electrolyte abnormalities [2,3]. These patients are at risk of developing both hyponatremia secondary to SIADH secretion and CSWS and hypernatremia secondary to diabetes insipidus. They may also have behavioral abnormalities that interfere with their treatment. This could represent new-onset schizophrenia or the effects of brain contusion. These abnormalities can lead to excessive water ingestion or inadequate water ingestion. The clinician should expect these patients to have abnormalities in electrolytes and order serial measurements of sodium. Patients with increases or decreases in sodium need a more comprehensive clinical and laboratory evaluation, and clinicians need to decide if the patient is euvolemic, hypovolemic, or hypervolemic. Urine output and urine electrolytes should be measured, and thyroid and adrenal function tests should be obtained. Patients with hyponatremia and clinical symptoms attributable to this hyponatremia should have their serum sodium levels corrected. This should be performed cautiously to avoid the possibility of osmotic demyelination [43]. Patients with hypernatremia will need volume expansion. Patients with abnormal thirst may need fluid restriction or scheduled fluid administration. Dysnatremia and fluid status are ongoing concerns and need frequent reevaluation. Comprehensive supportive care, including thiamine administration, is obviously essential [65]. All four sodium disorders can persist posthospitalization and complicate rehabilitation and chronic care [66].

This paper establishes a strong correlation between TBI and several sodium disorders (diabetes insipidus, SIADH, CSWS, and psychogenic polydipsia), but TBI is considered a single entity in this review. More specific analysis of clinical evidence on the impact of different injury mechanisms/types would be needed to answer questions about the type of injury and electrolyte disorders. However, some potential relationships based on the mechanisms discussed provide the basis for future clinical studies. Damage to the hypothalamus and pituitary glands, which regulate AVP secretion and thirst, is a key factor in sodium dysregulation. Therefore, TBI types that directly involve these brain regions would be expected to have a more significant impact. Penetrating injuries, for instance, could cause more focal damage to these regions, leading to different types of hormonal imbalances than diffuse injuries like concussion or blunt force trauma, but studies evaluating these possibilities would be extremely difficult to carry out and would require large databases with a prospective collection of information. In addition, the severity of the TBI, regardless of type, plays a significant role. More severe TBIs are more likely to cause hyponatremia or hypernatremia. The degree of hypothalamic–pituitary damage is likely proportional to injury severity, and a severely damaged system is more prone to dysregulation regardless of the specific mechanism of injury. However, this conclusion needs support from prospective studies using semiquantitative injury scores to classify patients into injury categories.

The management of these patients is complicated by several important and unavoidable uncertainties. Is the timeframe during which the patient develops either hyponatremia or hypernatremia important, and does it influence poor outcomes? Slow changes in sodium levels over hours and days are likely less consequential. Is the actual level of sodium important? Are small changes less important than large changes? Any change in sodium level should lead to a review of the patient’s clinical status. The patient’s clinical status may not be attributable to their current sodium level, given the fact that the patient has had a significant TBI. Corrections of sodium levels should begin immediately, but the rate of correction should be slow. Some patients have developed osmotic demyelination independent of changes in sodium concentrations. Finally, the clinician should not assume that the sodium level at discharge from the hospital is stable, and frequent measurements will be needed during posthospital rehabilitation and follow-up clinic visits.

In summary, abnormal sodium concentrations occur frequently in patients with TBI. It may be difficult to determine if the abnormal sodium concentration contributes to the clinical presentation, but the possibility of this should be an ongoing consideration. Treatment should aim for stable sodium levels in the normal range. This important clinical disorder needs more study.

The complex clinical disorders associated with traumatic brain injury make it difficult to determine the key factors that contribute to poor outcomes, such as mortality and chronic neurological disability. Prospective multicenter studies are essential to understand the relationship between sodium disorders and patient outcomes. Critical research to carry out includes analyzing the correlation between fluid balance and hospital mortality and assessing whether different types of fluid administration affect mortality rates [67]. Further investigation is needed into the stability of sodium levels during the first week of hospitalization and the potential utility of copeptin levels as a biomarker for neurohypophysis function, including how these levels correlate with patient outcomes [68]. In addition, copeptin levels in conjunction with serum sodium and urine sodium measurements can help establish the type of sodium disorder. It is possible that these studies could lead to changes in management protocols involving fluid administration and the rate of sodium correction if abnormalities are found. This information should be collected relatively quickly, given the high mortality rate of traumatic brain injuries, which makes study endpoints definitive. Ultimately, advances in understanding and managing fluid and sodium imbalances could significantly improve patient prognosis and care practices.

## Figures and Tables

**Table 1:** Diabetes insipidus and traumatic brain injury.

Frequency	Overall, 15–20% of patients with TBI develop DI. Exact figures are difficult to determine due to variations in diagnostic criteria and patient populations.
Pathophysiology Related to Trauma	Direct injury to the hypothalamic–pituitary axis, often involving the supraoptic and paraventricular nuclei. Disruption of AVP synthesis, transport, or release. Damage to the posterior pituitary gland. Edema or hemorrhage affecting the hypothalamic–pituitary axis. Indirect effects from elevated intracranial pressure or other TBI-related complications can disrupt water homeostasis.
Pathophysiology of Sodium Disorders	Deficiency or impaired action of the antidiuretic hormone (ADH) leads to decreased water reabsorption in the kidneys. This results in increased free water excretion and increased serum osmolality. Polyuria and polydipsia are classic symptoms resulting from the body’s attempt to restore fluid balance. Hypernatremia occurs from excessive water loss and is a serious clinical finding. Low urine osmolality is due to the inability of the kidney to concentrate in urine.
Laboratory	Hypernatremia (serum sodium > 145 mEq/L). Elevated serum osmolality (>295 mOsm/kg). Low urine osmolality (<300 mOsm/kg). Low copeptin levels (<5 pmol/L in central DI). A water deprivation test may be employed to differentiate central vs. nephrogenic DI. Hypertonic saline infusion or arginine vasopressin stimulation tests are more sensitive and specific.
Treatment	Desmopressin (synthetic AVP): oral, intranasal, subcutaneous, or intravenous administration. Fluid replacement to correct dehydration and hypernatremia. The treatment of underlying TBI-related complications. Management of associated behavioral or psychiatric problems such as psychogenic polydipsia. Close monitoring of fluid balance and serum electrolyte levels.
Associated Mortality	A ~60% mortality rate is reported in some studies (note: mortality is greatly influenced by the severity of both DI and the underlying TBI). This is a critically ill population, and other factors likely contribute to the high mortality.

AVP—arginine vasopressin; DI—diabetes insipidus; TBI—traumatic brain injury.

**Table 2:** Syndrome of inappropriate antidiuretic hormone in traumatic brain injury.

Frequency	Incidence varies widely across studies (10–40% in severe TBI). Exact figures are difficult to determine due to variations in diagnostic criteria and patient populations.
Pathophysiology Related to Trauma	TBI-induced disruption of the hypothalamic–pituitary axis. Potential direct injury to neurosecretory cells. Indirect effects via increased intracranial pressure (ICP). Disruption of the blood–brain barrier. Secondary effects from increased ICP, cerebral edema, or other TBI-related complications.
Pathophysiology of Sodium Disorder	Inappropriate ADH secretion or enhanced renal sensitivity to ADH. Excessive water reabsorption leads to dilutional hyponatremia. Impaired thirst mechanisms may exacerbate this condition.
Laboratory Findings	Hyponatremia (<135 mEq/L). Hypo-osmolality (<275 mOsm/kg). Hyperosmolar urine (>100 mOsm/kg). Normal or slightly increased urine sodium concentration. Euvolemia or mild hypervolemia. Low serum uric acid and urea nitrogen levels. Elevated copeptin levels may be present. The water deprivation test may be helpful in selected cases.
Treatment	Fluid restriction (often initial). Careful monitoring of serum sodium and neurological status. Gradual correction of hyponatremia to prevent ODS. Vaptans (ADH receptor antagonists) may be considered. Supportive care for other TBI complications.
Associated Mortality	Increased mortality is associated with severe SIADH in TBI. Mortality risk varies with SIADH and TBI severity. Precise figures are difficult to obtain due to the variability in study populations and diagnostic criteria.

SIADH—syndrome of inappropriate antidiuretic hormone; TBI—traumatic brain injury; ADH—antidiuretic hormone.

**Table 3:** Cerebral salt-wasting syndrome from traumatic brain injury.

Frequency	Incidence varies significantly across studies (reported as high as 40% in severe TBIs in some studies, but often lower). Prevalence likely depends on TBI severity, diagnostic criteria, and patient populations. Distinguishing CSWS from SIADH can be challenging due to overlapping clinical features and the need to consider volume status. Precise diagnostic criteria are not universally agreed upon.
Pathophysiology Related to Trauma	Thought to be caused by disruption to the blood–brain barrier, leading to excessive sodium loss in urine. Possibly related to elevated intracranial pressure, brain edema, and/or the activation of natriuretic peptides. Mechanisms underlying CSWS in TBI are not fully understood and are likely multifactorial. They may involve direct injury to brain regions involved in sodium regulation.
Pathophysiology of Sodium Disorder	Excessive renal sodium wasting despite euvolemia or hypovolemia, resulting in hyponatremia and hypovolemia. Often associated with persistent natriuresis. Characterized by a low effective circulating volume despite relatively normal total body sodium.
Laboratory	Hyponatremia (<135 mEq/L) Hypovolemia (low blood pressure, tachycardia, elevated BUN/creatinine) High urine sodium excretion (>20–40 mEq/L). Urine osmolality is inappropriately low, with plasma osmolality often normal or slightly decreased. Serum uric acid may be elevated.
Treatment	Fluid resuscitation (isotonic saline is usually preferred). Sodium supplementation (oral or intravenous). Mineralocorticoids (fludrocortisone) in selected cases can improve sodium retention. Treatment of underlying TBI-related complications.
Associated Mortality	Increased mortality is associated with CSWS in TBI, likely due to the severity of the underlying TBI, complications of hyponatremia, and treatment challenges. Precise figures are difficult to obtain due to variations in study populations and diagnostic criteria.

TBI—traumatic brain injury; CSWS—cerebral salt-wasting syndrome.

**Table 4:** Thirst in traumatic brain injury.

Frequency	Variable, often not directly assessed; associated with hyponatremia/hypernatremia. Related to psychiatric conditions like chronic schizophrenia in up to 11–20% of cases, but unclear data with other psychiatric conditions.
Pathophysiology Related to Trauma	Direct injury to the hypothalamic thirst center. Damage to osmoreceptors. Disruption of neural pathways involved in thirst regulation. Elevated intracranial pressure and cerebral edema may impair thirst sensation or response. Secondary psychological factors, including delirium or depression, can also alter thirst perception or drinking behavior.
Pathophysiology of Sodium Disorder	Abnormal thirst can lead to either excessive water intake (psychogenic polydipsia) resulting in hyponatremia or inadequate fluid intake resulting in hypernatremia (particularly in association with diabetes insipidus). The resulting sodium imbalances cause severe clinical sequelae, including neurologic dysfunctions and elevated intracranial pressure.
Laboratory	Serum sodium levels (hyponatremia or hypernatremia). Serum and urine osmolality with urine volume and sodium excretion. The assessment of volume status (euvolemia, hypovolemia, hypervolemia).
Treatment	Treatment depends on the underlying cause of thirst dysfunction and the presence of associated sodium imbalances. Psychogenic polydipsia may require behavioral interventions, the measurement of diurnal weight, and fluid restriction in periods between 1 and 3 h within a supervised setting. Desmopressin is a mainstay of treatment for diabetes insipidus, but it is not appropriate if fluid intake is the issue. The careful monitoring of fluid balance, serum sodium levels, and neurological status.
Associated Mortality	Increased mortality risks associated with severe fluid imbalances, particularly hypernatremia. This is likely to be greatly influenced by other underlying TBI-related pathologies. Precise mortality figures are challenging to obtain due to the difficulties in assessing the contribution of thirst to overall morbidity and mortality.

## Data Availability

All information in this review was recovered from the references used for the review.
